# The Effect of Plant-Based Protein Ingestion on Athletic Ability in Healthy People—A Bayesian Meta-Analysis with Systematic Review of Randomized Controlled Trials

**DOI:** 10.3390/nu16162748

**Published:** 2024-08-17

**Authors:** Shiao Zhao, Yipin Xu, Jiarui Li, Ziheng Ning

**Affiliations:** Faculty of Health Sciences and Sports, Macao Polytechnic University, R. de Luís Gonzaga Gomes, Macao 999078, China; p2314214@mpu.edu.mo (Y.X.); p2313773@mpu.edu.mo (J.L.); zhning@mpu.edu.mo (Z.N.)

**Keywords:** plant-based protein, athletic performance, macronutrients, muscle protein synthesis, endurance ability, muscle strength

## Abstract

Plant-based protein supplements are increasingly popular, yet their efficacy in enhancing athletic performance compared to animal protein, insect protein, or other protein types remains under investigation. This study aimed to assess the effectiveness of plant-based protein on athletic abilities such as muscle strength, endurance performance, and muscle protein synthesis (MPS) rate and compare it to no- or low-protein ingestion and non-plant protein sources. Randomized controlled trials (RCTs) evaluating the beneficial and harmful effects of plant-based protein ingestion on athletic ability in healthy individuals were considered. A systematic search of six databases yielded 2152 studies, which were screened using the Covidence systematic review tool. Thirty-one studies were included for meta-analysis after independent selection, data extraction, and risk of bias assessment by two reviewers. The meta-analysis employed a Bayesian approach using the Markov chain Monte Carlo (MCMC) method through a random-effects model. The results demonstrated that plant-based protein supplements provided greater benefits for athletic performance in healthy individuals compared to the no- or low-protein ingestion group [μ(SMD): 0.281, 95% CI: 0.159 to 0.412; heterogeneity τ: 0.18, 95% CI: 0.017 to 0.362]. However, when compared to other types of protein, plant-based protein ingestion was less effective in enhancing athletic ability [μ(SMD): −0.119, 95% CI: −0.209 to −0.028; heterogeneity τ: 0.076, 95% CI: 0.003 to 0.192]. A subgroup analysis indicated significant improvements in muscle strength and endurance performance in both young and older individuals consuming plant-based protein compared to those with no- or low-protein ingestion. Nonetheless, other protein types showed greater benefits in muscle strength compared to plant-based protein [μ(SMD): −0.133, 95% CI: −0.235 to −0.034; heterogeneity τ: 0.086, 95% CI: 0.004 to 0.214]. In conclusion, while plant-based protein ingestion demonstrates superior efficacy compared to low- or no-protein ingestion, it is not as effective as other protein types such as whey, beef, or milk protein in enhancing athletic performance in healthy individuals. Registration: Registered at the International Prospective Register of Systematic Reviews (PROSPERO) (identification code CRD42024555804).

## 1. Key Points

Plant-based protein could improve athletic performance and MPS in healthy people compared to no- or low-protein ingestion.

Plant-based protein could not provide greater gains in improving MPS and athletic performance, including muscle strength and endurance performance, compared to other types of protein.

Plant-based protein seemed to be less effective than other types of protein in some outcomes.

## 2. Background

In the fast-developing world, nutrition and diet have garnered increasing attention, particularly in sports-related areas aimed at enhancing health and achieving optimal body composition. Appropriate diet control and supplement ingestion can significantly improve athletic ability, prevent disease, and reduce body fat proportion. As a critical macronutrient, protein plays a vital role in human health; however, the efficacy of protein ingestion on athletic performance, especially plant-based protein ingestion, remains ambiguous and controversial. According to the National Strength and Conditioning Association’s (NSCA) guide to sport and exercise nutrition, soy protein is a high-quality, complete protein. Its Protein Efficiency Ratio (PER) and Protein Digestibility-Corrected Amino Acid Score (PDCAAS) are comparable to those of dietary meat or fish and slightly lower than those of egg, milk, casein, bovine colostrum, and whey protein, making soy protein supplementation a viable choice for people [1].

Research on the effects of plant-based protein ingestion on athletic ability, including muscle strength, endurance performance, and muscle protein synthesis (MPS), is scarce, and its benefits remain unclear. Existing studies have produced mixed results. Some studies have demonstrated that plant-based proteins can be as effective as other protein types in enhancing athletic ability in healthy individuals [2,3,4,5,6]. For instance, Loureiro et al. compared pea protein and whey protein, highlighting the viability of plant protein as an alternative to animal protein without compromising athletic performance or recovery [7]. Additionally, some authors have found a strong association between plant protein ingestion and improved athletic ability. Goash et al. concluded that soy protein combined with sago co-ingestion significantly improved endurance performance and reduced post-exercise fatigue [8]. Moreover, plant protein ingestion has been shown to enhance muscle strength in both trained and untrained individuals [9,10].

Plant-based proteins are high-quality supplements that can augment MPS in both males and females [11,12,13,14,15]. For example, Mckendry et al. found that ingesting plant-based protein after breakfast and lunch enhanced MPS in older males [16]. Similarly, Li et al. concluded that increasing dietary protein intake, regardless of its source, could be beneficial for preserving skeletal muscle mass [17]. Conversely, Stephan et al. reported that soy protein consumption resulted in lower MPS rates compared to whey, milk, or beef protein [18]. Reviews have also indicated that vegetable protein supplementation can provide similar ergogenic effects to animal proteins, such as increased muscle strength, improved MPS, and reduced body fat mass [19]. Pinckaers et al. found that wheat protein could improve MPS in healthy and young males, but there was no difference between milk protein, wheat protein, and protein blend supplements [20]. Despite these findings, the relationship between plant-based protein and MPS remains inconclusive, necessitating further research.

Contrary to these positive findings, some studies suggest that plant-based proteins offer limited benefits for athletic performance. For instance, Wirth et al. observed no significant differences in muscle function, body composition, metabolic health, sleep quality, or quality of life after a 12-week intervention of increased protein intake (both plant-based and dairy-based) compared to a low-protein group [21]. Reidy et al. reported that plant-based protein supplementation slightly enhanced gains in lean body mass but did not improve strength gains in healthy males [22]. Furthermore, recent studies on soccer players have shown that neither plant-based nor whey protein supplementation significantly impacted athletic performance, including endurance and muscle strength [7,23]. Aside from these, multiple studies have stated that plant protein cannot improve endurance performance and may even impair gains in muscle strength in healthy individuals [24,25,26].

Additionally, plant-based proteins appear to have different effects on young and older individuals. While soy protein ingestion combined with resistance training improved body composition and metabolic function in middle-aged males [27], other studies have found no significant differences in muscle function and metabolic health in older individuals [21]. Thomson et al. noted that increased soy protein intake attenuated gains in muscle strength during resistance training in older adults compared to dairy protein or usual protein intake [23]. 

Despite these varying viewpoints, pea protein is recognized as a promising supplement for supporting muscle protein synthesis and exercise performance, warranting further research to determine how it compares with animal proteins [28,29]. Pea protein has also shown effectiveness in reducing muscle damage and enhancing muscle recovery [30]. Therefore, this study aimed to investigate the efficacy of plant-based protein. This study employs a Bayesian meta-analysis to quantitatively support these conclusions.

The objective of this study is to investigate the efficacy of plant-based protein on athletic ability in healthy individuals, including both young and older populations. It is hypothesized that plant-based protein will have a beneficial effect on athletic ability, encompassing muscle strength, endurance performance (both aerobic and anaerobic), and muscle protein synthesis.

## 3. Methods

This study was registered in PROSPERO (CRD42024555804) and reported in accordance with PRISMA guidelines (see Supplementary Materials). A Bayesian meta-analysis with a systematic review was conducted using Covidence, Stata, GRADEprofiler, R, Review Manager, and Get Data Digitizer.

### 3.1. Search Strategy

A comprehensive search strategy was developed using Medical Subject Headings (MeSH) and free-text search terms to systematically screen the EBSCO, PubMed, Ovid, Web of Science, ProQuest, and Scopus databases. A total of 2152 studies were extracted by two authors using the online tool Covidence for systematic review. The keywords and subject headings were confirmed through discussion between the two authors. The confirmed search terms included: ‘Soy protein OR plant protein OR plant-based protein OR pea protein OR peanut protein OR potato protein OR plant protein supplements AND healthy adults AND post-exercise recovery OR athletic performance OR sports performance OR muscle strength OR resistance training OR endurance performance OR aerobic ability OR muscle protein synthesis OR anaerobic ability OR lower body strength OR upper body strength’. These terms were used across all specified databases. The exact search strategy in each database (Table S1) can be seen in Supplementary Materials.

### 3.2. Inclusion and Exclusion Criteria

Following the PICOS principle, non-human studies and non-comparative studies were excluded. Eligible studies were randomized controlled trials (RCTs) that included plant-based protein diets or supplements. Studies with a mixture of multiple protein types were excluded. Participants had to be healthy individuals, aged 16 or above, and studies involving patients or obese populations were excluded. Non-original studies such as reviews, letters, or editorials were excluded, as well as studies lacking extractable data related to exercise or athletic ability.

Both parallel and crossover RCTs were included. Participants could be of any gender, and the experimental group involved plant-based protein diets or supplements, while the control group involved no or low protein or other types of protein. Outcomes had to be related to athletic ability.

### 3.3. Selection Process

**Figure 1:**
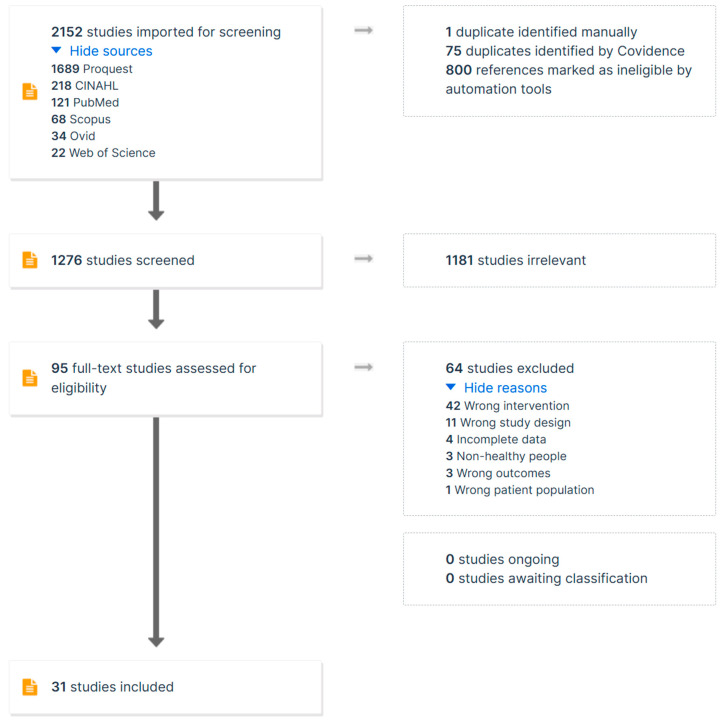
PRISMA flow chart for the identification of the included studies.

The selection process and information sources are illustrated in Figure 1. Two reviewers (S.Z. and Y.X.) independently screened titles and abstracts, followed by full texts, against the eligibility criteria using Covidence. When conflicts arose, a third and a fourth reviewer (R.L. and Z.N.) were invited to discuss the solution and revised the selection results in Covidence. Covidence automatically excluded 75 duplicate studies, and 1 duplicate was excluded manually. A total of 2152 studies were screened, with 800 marked as ineligible by the automatic tool and 1181 excluded manually as irrelevant. After full-text screening of 95 studies, 64 were excluded, leaving 31 studies included in the meta-analysis.

### 3.4. Risk of Bias Assessment

The risk of bias for all included studies was independently assessed using the guidelines and criteria outlined in the Cochrane Handbook for Systematic Reviews of Interventions. Two authors (S.Z. and Y.X.) assessed the included studies through the Cochrane risk of bias (ROB) criteria in RCTs within Covidence. Seven areas of bias were evaluated: (1) random sequence generation; (2) allocation concealment; (3) blinding of participants and personnel; (4) blinding of outcome assessment; (5) incomplete outcome data; (6) selective reporting; and (7) other bias. The risk of bias was classified as low, unclear, or high. After independent assessments, the authors reached a consensus through discussion. The final results were recorded in an Excel 365 template and input into R software to create risk of bias summary plots using the Robvis and Ggplot2 R packages. Studies with more than two and fewer than four areas marked as unclear risk were classified as moderate risk overall.

Additionally, Bayesian funnel plots were generated using the Bayesmeta R package [31] to check the symmetry of the included data, represented as circle dots distributed on both sides of the funnel plots.

### 3.5. Certainty in Evidence

GRADEprofiler 3.6 software was used to assess each result. The quality of the evidence regarding plant-based protein was assessed using the GRADE approach, which provides a transparent method to rate the quality of evidence across studies by evaluating risk of bias, inconsistency of results, indirectness, and imprecision of effect estimates. The GRADE approach classifies the quality of evidence as high, moderate, low, or very low.

### 3.6. Data Extraction

Data were extracted independently by two authors (S.Z. and Y.X.) using Covidence, with conflicts resolved through discussion with the third and fourth authors (R.L. and Z.N.). For each study, characteristics such as intervention description, first author, publication year, study design, country, participants’ ages, BMI, plant-based protein type, protein intake dosage, duration, and outcome data type were extracted. The outcomes included time to exhaustion, lower body strength, upper body strength, 1RM, cycling time trials, maximum voluntary contraction (MVC), counter-movement jump (CMJ), muscle protein synthesis rate, anaerobic peak and average power, vertical jump, cycling distance, hand grip strength, maximum speed, average speed, and Vo_2max_. Data were presented as mean plus standard deviation (M ± SD). Review Manager was used to convert data not initially in M ± SD format.

When data were not presented as exact numbers, Get Data Digitizer [31] was used to extract data from graphs. All data measured in the 31 studies were classified into two types: mean change difference with corresponding standard deviation (ΔSD) to compare intervention changes between groups and final values after intervention to compare differences between groups. When ΔSD was not reported, it was estimated using the correlation coefficient (corr) formula provided by the Cochrane Handbook for meta-analysis of intervention:

corr = (SDpre^2^ + SDpost^2^ − SDchange^2^)/(2 × SDpre × SDpost)

The ΔSD was then calculated using the following formula:

∆SD = √(SDpre^2^ + SDpost^2^ − 2 × corr × SDpre × SDpost)

### 3.7. Summary Measures and Synthesis

Two comparisons were classified for meta-analysis: (1) plant-based protein group vs. no- or low-protein group and (2) plant-based protein group vs. other types of protein group.

A meta-analysis using Bayesian and traditional frequentist methods was performed on 31 RCTs in Rstudio 1.2.5019. The frequentist meta-analysis used Stata 17 and Review Manager 5.3 software to assess. The Bayesian meta-analysis used the Bmeta and Metafor escalc R packages to calculate effect size (SMD) and variance reciprocal in each study. The Bayesian approach is considered suitable for meta-analyses including few studies, providing evidence for both null and alternative hypotheses, and offering complete information about credible parameter values and the probability of any given value [32,33,34,35,36]. 

Continuous data were expressed as standardized mean deviations with 95% credible intervals. Pooled estimates were calculated using the random-effects model to account for inevitable heterogeneity between the included studies. A Markov chain Monte Carlo (MCMC) sampler with three chains was used, and heterogeneity was assessed by analyzing τ. Non-informative prior distributions were used to maximize information due to the lack of empirically based prior distributions [35,37]. Trace plots and ergodic mean plots generated by the Mcmcplots R package were used to confirm the convergence of the Markov chain, ensuring the reliability of results and parameters. Traditional frequentist analyses were also conducted for comparison and sensitivity analysis.

There are two signs using blue square and red circle to represent the random-effects model and no-pooling effects model. But, the circle sign were transformed to diamond because of the too little space and too much data in some Bayesian forest plots. The red diamond also represented the no-pooling effects model, and there was no difference between red circle and diamond.

### 3.8. Subgroup Analysis

Subgroup analyses were conducted based on age and type of athletic performance. Participants were classified as older (age > 50 years) or younger (age < 50 years). Athletic performance was divided into a muscle strength group and an endurance performance group for further analysis.

## 4. Results

### 4.1. Study Characteristics

All studies included in this meta-analysis were randomized controlled trials (RCTs). Eight studies were crossover designs [7,8,13,22,26,38,39,40], while twenty-three employed parallel designs. The detailed characteristics of the 31 included studies are summarized in Table 1 and Table 2. The meta-analysis encompassed 1116 participants, with 799 males and 227 females; two studies did not report the participants’ sex [23,41]. The mean age of the participants ranged from 17 to 66.5 years, with the majority in the 17–32 age group (68%), followed by the 56–67 age group (32%). Most studies originated from Europe and North Africa (97%); one study originated from Asia (3%) [8] and one from Australia [23].

The plant-based proteins studied included soy or pea protein in 20 studies (65%), plant protein mixtures in 5 studies (16%), wheat protein in 1 study [22], potato protein in 2 studies [11,12], corn protein in 1 study [42], and mung bean and fava bean protein in 2 studies [9,43]. 

### 4.2. Risk of Bias of Included Studies

**Figure 2:**
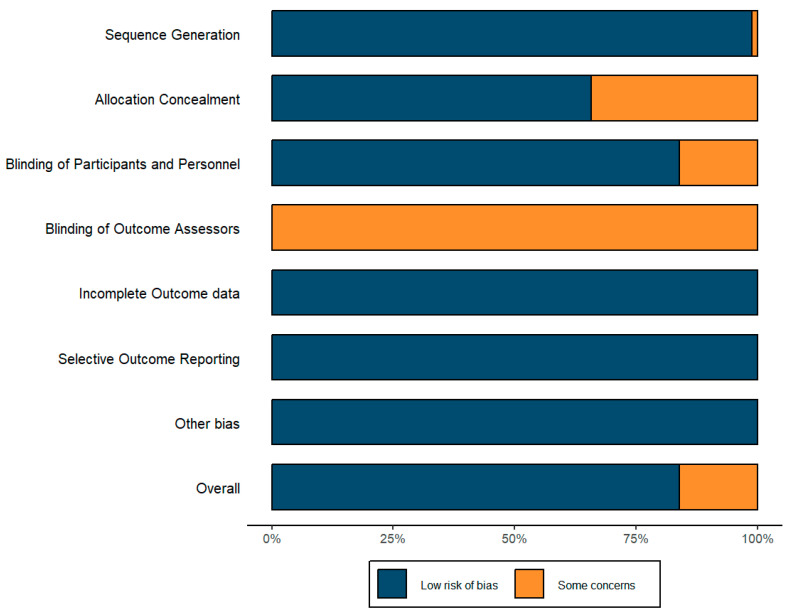
Risk of bias summary.

**Figure 3:**
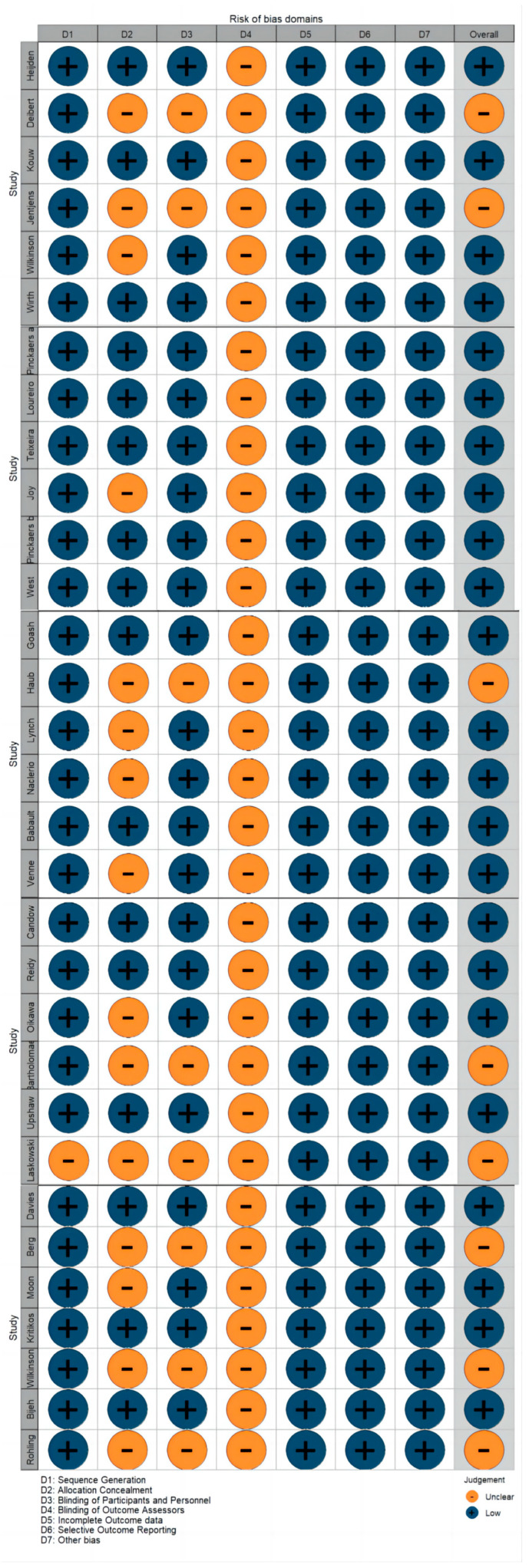
Risk of bias graph.

The risk of bias assessment details are presented in Figure 2 and Figure 3. The Cochrane risk of bias scale (ROB) was utilized to assess the included studies, with results visualized through the Robvis and Ggplot2 R packages. No study was marked as high risk in any area. Some studies did not provide clear information on blinding of outcome assessors (29%) and allocation concealment (52%), and one study lacked sufficient details on sequence generation [41]. These areas were marked as unclear risk. Overall, over 75% of the studies were assessed as low risk of bias and less than 25% as moderate risk.

### 4.3. Quality Grade in Each Outcome

**Figure 4:**
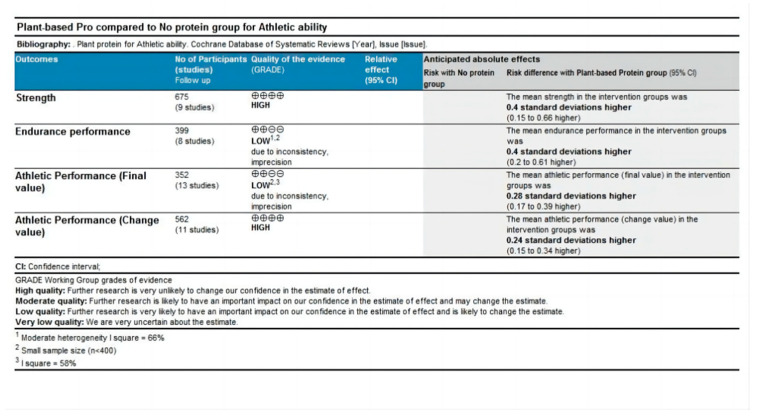
Quality grade of athletic ability (plant-based protein vs. no protein).

**Figure 5:**
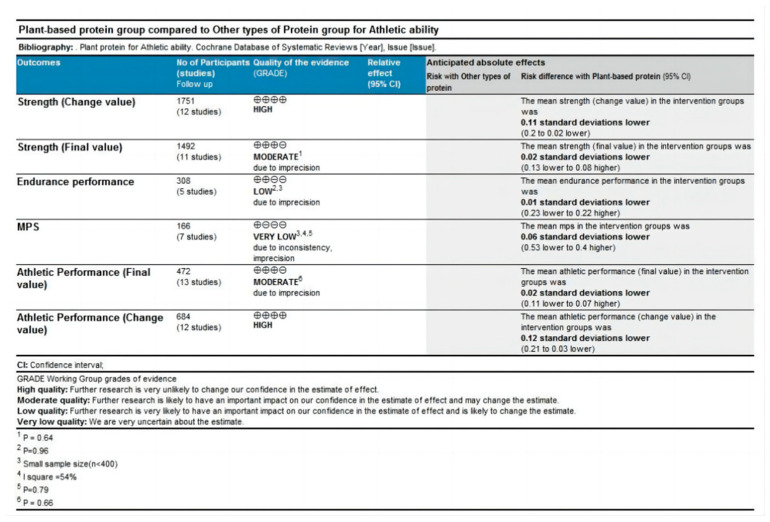
Quality grade of athletic ability (plant-based protein vs. other types of protein).

Data from ten outcomes across two comparisons (plant-based protein vs. no protein and plant-based protein vs. other types of protein) were assessed (Figure 4 and Figure 5). For the plant-based protein vs. no protein comparison (Figure 4), endurance performance and athletic performance outcomes presented by final value were rated as low grade of evidence due to moderate heterogeneity and small sample size. Strength and athletic performance outcomes presented by change value were rated as high grade of evidence. For the plant-based protein vs. other types of protein comparison (Figure 5), strength and athletic performance outcomes presented by final value were rated as moderate grade of evidence due to statistical insignificance. Muscle protein synthesis (MPS) was rated as very low grade of evidence due to small sample size, moderate heterogeneity, and statistical insignificance. Endurance performance was rated as low grade of evidence for similar reasons. Strength and athletic performance outcomes presented by change values were rated as high grade of evidence.

### 4.4. Convergence of the Markov Chain

**Figure 6:**
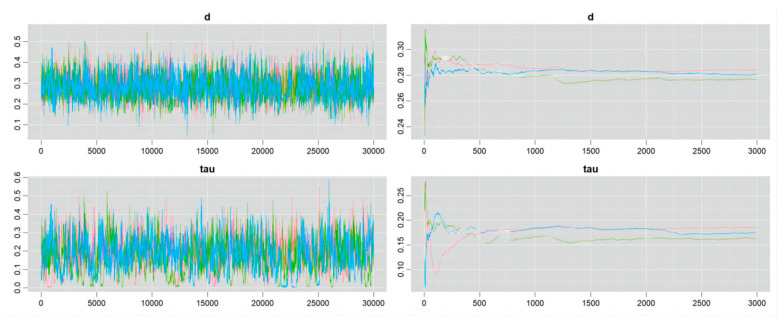
Convergence of Markov chain in the outcome of athletic performance (change value, plant-based protein vs. no protein).

**Figure 7:**
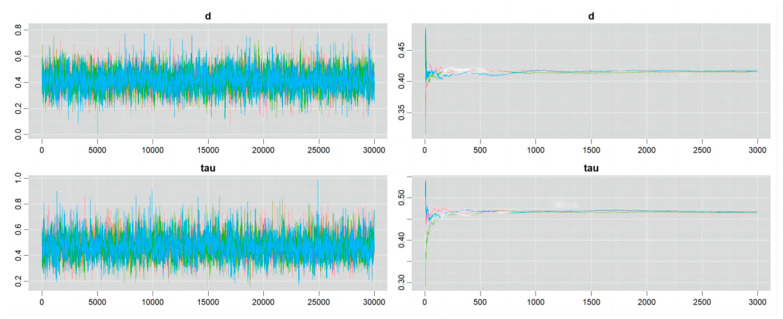
Convergence of Markov chain in the outcome of athletic performance (final value, plant-based protein vs. no protein).

**Figure 8:**
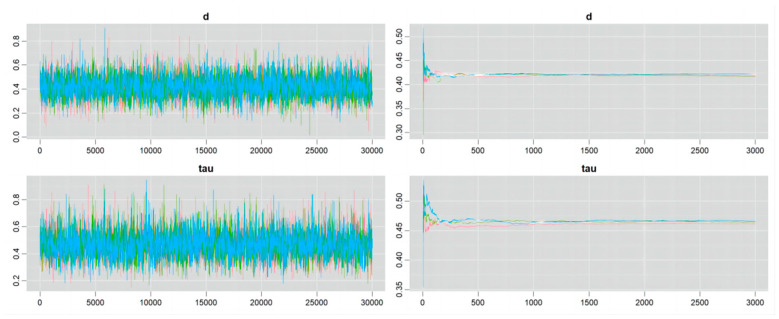
Convergence of Markov chain in the outcome of athletic performance (change value, plant-based protein vs. other types of protein).

**Figure 9:**
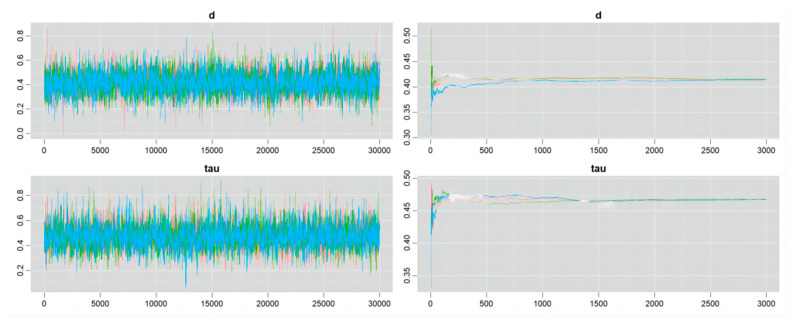
Convergence of Markov chain in the outcome of athletic performance (final value, plant-based protein vs. other types of protein).

Details of the Markov chain convergence are shown in Figure 6, Figure 7, Figure 8 and Figure 9. The ergodic mean was stable in each plot, and the parameters of d and tau exhibited minor fluctuations around their respective means in each trace plot, indicating credible results from the Bayesian meta-analysis.

### 4.5. Meta-Analysis

#### 4.5.1. Results of Plant-Based Protein vs. No Protein

Twenty-four studies compared the effect of plant-based protein vs. no protein on athletic performance. The summary of the Bayesian and frequentist meta-analysis results for two outcomes is presented in Table 3. Each included studies had different data like muscle strength, endurance performance or etc. The English letters or English lerrers combined with numbers represented different data in a same study, like “Bijeh a” and “Bijeh a^1^”.

**Figure 10:**
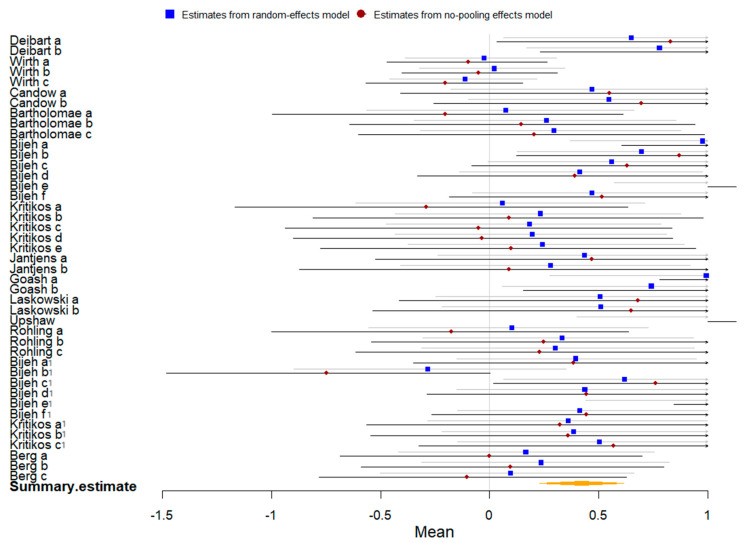
Bayesian forest plot of athletic performance (final value, plant-based protein vs. no protein).

Thirteen studies involving 352 participants were included in the meta-analysis of athletic performance presented by final value. The Bayesian meta-analysis (Figure 10) showed a statistically significant effect [μ(SMD): 0.418, 95% CI: 0.229 to 0.611], with moderate heterogeneity (τ: 0.467, 95% CI: 0.283 to 0.684), Rhat = 1.001, and DIC = 103.2. The frequentist meta-analysis yielded an effect size estimate of 0.28 [95% CI: 0.17 to 0.39, *p* < 0.00001, *I*^2^ = 58%, Z = 4.9], indicating no significant difference from the Bayesian results.

**Figure 11:**
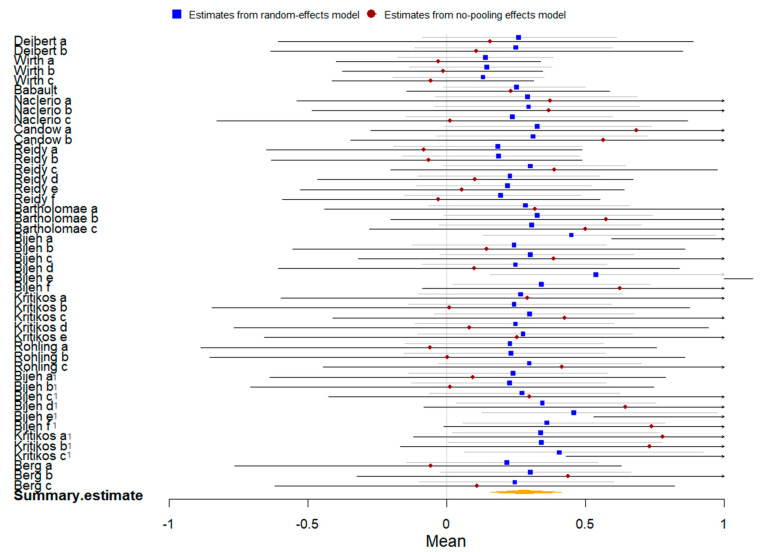
Bayesian forest plot of athletic performance (change value, plant-based protein vs. no protein).

Eleven studies with 562 participants were included in the meta-analysis of athletic performance presented by change value. The Bayesian meta-analysis (Figure 11) showed a statistically significant effect [μ(SMD): 0.281, 95% CI: 0.159 to 0.412], with low heterogeneity (τ: 0.18, 95% CI: 0.017 to 0.362), Rhat = 1.001, and DIC = 77.3. The frequentist meta-analysis yielded an effect size estimate of 0.24 [95% CI: 0.15 to 0.34, *p* < 0.00001, *I*^2^ = 24%, Z = 4.85], consistent with the Bayesian results.

**Figure 12:**
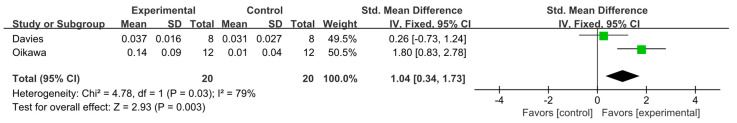
Frequentist forest plot of muscle protein synthesis (change value, plant-based protein vs. no protein).

Only two studies (40 participants) were included in the meta-analysis of muscle protein synthesis. A frequentist meta-analysis was performed, showing an effect size estimate of 1.04 [95% CI: 0.34 to 1.73, *p* = 0.003, *I*^2^ = 79%, Z = 2.93] (Figure 12).

#### 4.5.2. Results of Plant-Based Protein vs. Other Types of Protein

Fifteen studies compared plant-based protein vs. other types of protein on athletic performance, and seven studies compared them on muscle protein synthesis. The summary of the Bayesian and frequentist meta-analysis results for three outcomes is presented in Table 4.

**Figure 13:**
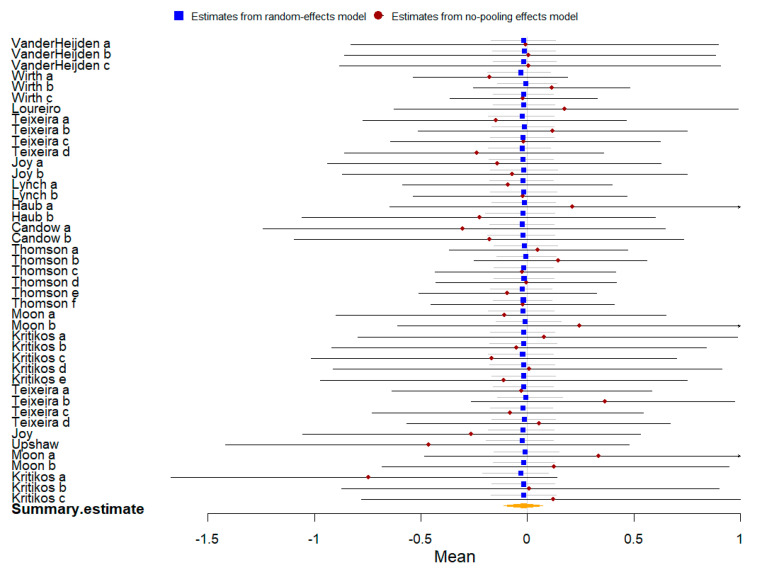
Bayesian forest plot of athletic performance (final value, plant-based protein vs. other types of protein).

Thirteen studies with 472 participants were included in the meta-analysis of athletic performance presented by final value. The Bayesian meta-analysis (Figure 13) showed no statistically significant effect [μ(SMD): −0.021, 95% CI: −0.118 to 0.072], with low heterogeneity (τ: 0.046, 95% CI: 0.001 to 0.128), Rhat = 1.003, and DIC = 1.8. The frequentist meta-analysis yielded an effect size estimate of −0.02 [95% CI: −0.11 to 0.07, *p* = 0.66, *I*^2^ = 0%, Z = 0.44], consistent with the Bayesian results.

**Figure 14:**
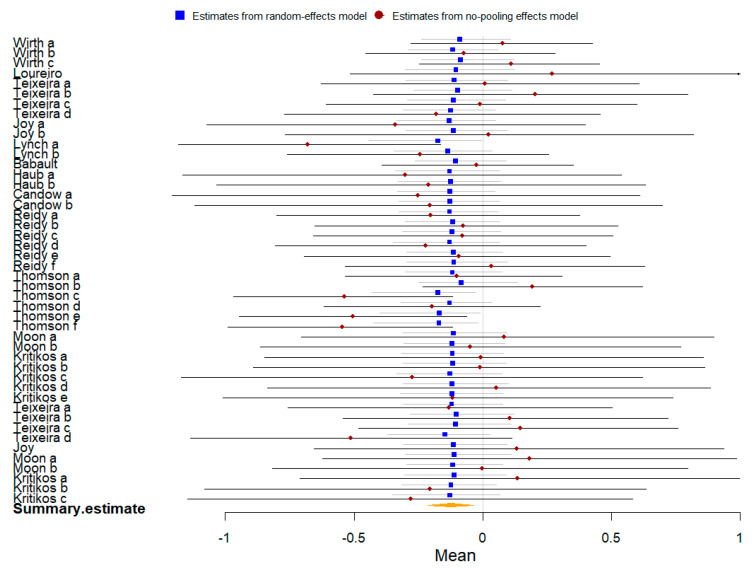
Bayesian forest plot of athletic performance (change value, plant-based protein vs. other types of protein).

Twelve studies with 684 participants were included in the meta-analysis of athletic performance presented by change value. The Bayesian meta-analysis (Figure 14) showed a small statistically significant effect [μ(SMD): −0.119, 95% CI: −0.209 to −0.028], with low heterogeneity (τ: 0.076, 95% CI: 0.003 to 0.192), Rhat = 1.003, and DIC = 16.2. The frequentist meta-analysis yielded an effect size estimate of −0.12 [95% CI: −0.21 to −0.03, *p* = 0.006, *I*^2^ = 0%, Z = 2.76], consistent with the Bayesian results.

**Figure 15:**
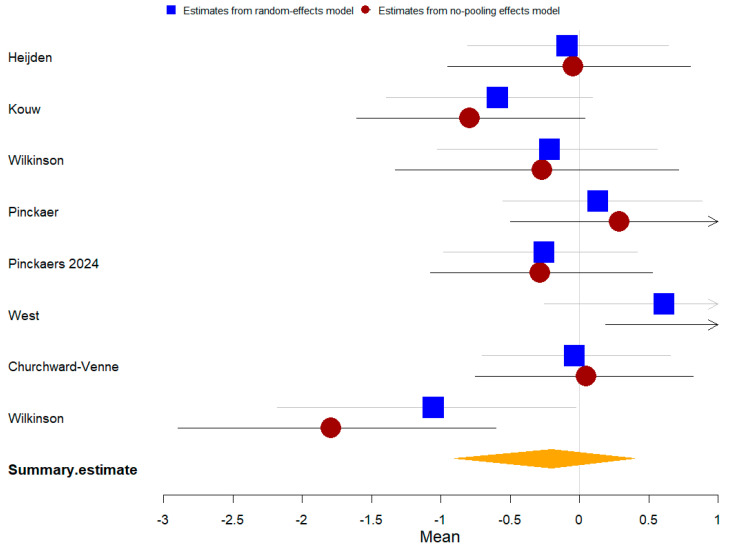
Bayesian forest plot of muscle protein synthesis (change value, plant-based protein vs. other types of protein).

Seven studies with 166 participants were included in the meta-analysis of muscle protein synthesis presented by change value. The Bayesian meta-analysis (Figure 15) showed no statistically significant effect [μ(SMD): −0.177, 95% CI: −0.866 to 0.491], with low heterogeneity (τ: 0.743, 95% CI: 0.116 to 1.704), Rhat = 1.001, and DIC = 22. The frequentist meta-analysis yielded an effect size estimate of −0.06 [95% CI: −0.53 to 0.4, *p* = 0.79, *I*^2^ = 54%, Z = 0.26], consistent with the Bayesian results.

#### 4.5.3. Subgroup Analysis

The subgroup analysis was divided into two parts: (1) the subgroup analysis based on the types of athletic performance and (2) the subgroup analysis based on age (age > 55 or <55). The subgroup analysis based on age aimed to explore the moderate heterogeneity (*I*^2^ = 58%) of athletic performance presented by final value in the meta-analysis comparing plant-based protein and no protein.

#### 4.5.4. Subgroup Analysis Based on Types of Athletic Performance

Four outcomes, including muscle strength and endurance performance, compared plant-based protein to no protein. The summary of the Bayesian and frequentist subgroup meta-analysis results is presented in Table 5. The Bayesian forest plots (Figures S1–S4) can be seen in Supplementary Materials.

Four outcomes, including muscle strength and endurance performance, compared plant-based protein to other types of protein. The summary of the Bayesian and frequentist subgroup meta-analysis results for four outcomes can be seen in Table 6. The Bayesian forest plots (Figures S5–S8) can be seen in Supplementary Materials.

In the comparison between plant-based protein and no protein, the plant-based protein group showed statistically significant improvements in muscle strength and endurance performance.

In the comparison between plant-based protein and other types of protein, the other types of protein group had statistical significance in the muscle strength presented by change value. The other three outcomes would not find any statistical significance in either the plant protein group or the other types of protein group. The effect of plant-based protein on athletic performance was similar to, and may even be less effective than, the intake of other types of protein.

#### 4.5.5. Subgroup Analysis Based on Age

This analysis aimed to explore the moderate heterogeneity (*I*^2^ = 58%) of athletic performance presented by final value in the meta-analysis comparing plant-based protein to no protein. Four outcomes of athletic performance were analyzed based on age (age < 55 years or >55 years). The summary of the Bayesian and frequentist subgroup meta-analysis results is presented in Table 7. The Bayesian forest plots (Figures S9–S12) can be seen in Supplementary Materials.

The results indicated high heterogeneity in the older age group’s meta-analysis. Excluding data from three studies involving older participants (>55 years) reduced this heterogeneity, suggesting that these studies contributed to the moderate heterogeneity observed.

### 4.6. Risk of Bias (Funnel Plots)

#### 4.6.1. Results of Plant-Based Protein vs. No Protein

**Figure 16:**
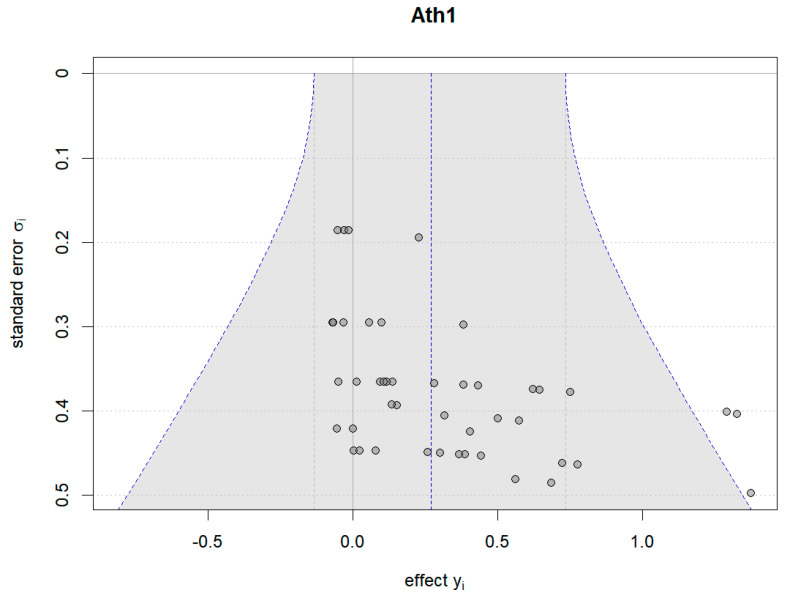
Funnel plot of athletic performance (change value).

**Figure 17:**
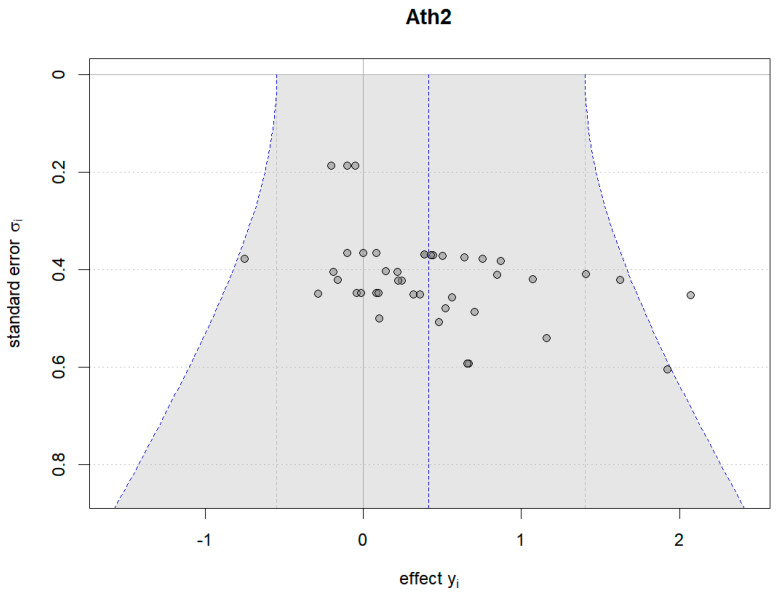
Funnel plot of athletic performance (final value).

Figure 16 and Figure 17 illustrate the Bayesian funnel plots assessing the risk of bias. Symmetry in the funnel plots indicates low or no risk of bias.

#### 4.6.2. Results of Plant-Based Protein vs. Other Types of Protein

**Figure 18:**
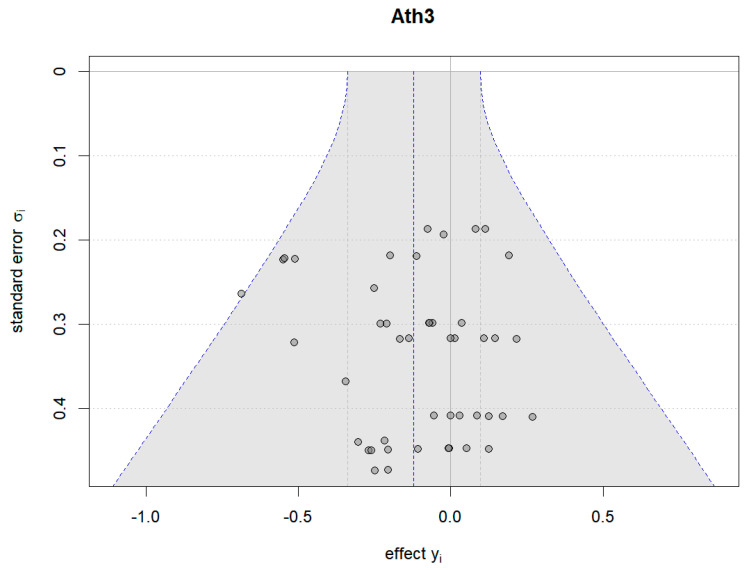
Funnel plot of athletic performance (change value).

**Figure 19:**
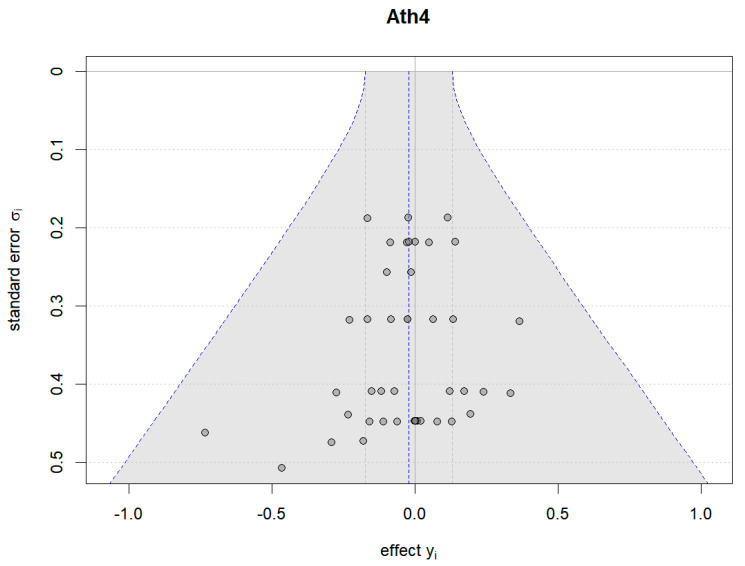
Funnel plot of athletic performance (final value).

**Figure 20:**
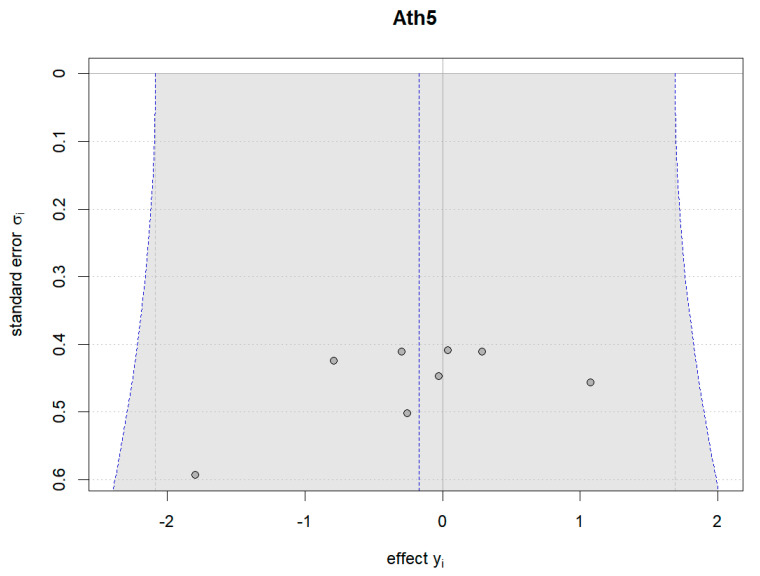
Funnel plot of muscle protein synthesis (change value).

Figure 18, Figure 19 and Figure 20 show the Bayesian funnel plots assessing the risk of bias in comparisons between plant-based protein and other types of protein. Symmetry in the funnel plots indicates low or no risk of bias. The funnel plot for muscle protein synthesis includes data from seven studies, and the limited number of studies may affect the accuracy of the risk of bias assessment.

## 5. Discussion

The current systematic review and meta-analysis summarize the evidence on the effect of (1) plant-based protein vs. no protein on athletic ability, including muscle strength, endurance performance, and muscle protein synthesis (MPS), as well as (2) plant-based protein vs. other types of protein on athletic ability, encompassing muscle strength, endurance performance, and MPS.

### 5.1. Plant-Based Protein vs. No Protein

This meta-analysis demonstrates that plant-based protein is superior to no-protein diets or supplements in enhancing athletic ability, including muscle strength, endurance performance, and MPS in healthy individuals. Various studies support these findings. Fritz et al. concluded that vegan protein ingestion improves muscle protein synthesis and skeletal muscle mass post-exercise [44]. The improvement in muscle strength and mass may be linked to anabolic hormone changes. Amino acids in soy protein, such as arginine and lysine, might influence the somatotropic axis and promote HGH release and its anabolic action [27]. While our study could not conclusively demonstrate these hormonal changes, plant-based proteins like soy and pea have been shown to improve muscle strength and mass [9,10,38,45], making them suitable choices compared to no-protein or low-protein supplements [19]. Subali et al. concluded that soy-based tempeh, rich in amino acids and L-arginine, is a promising vegan protein source for athletes, enhancing muscle strength and endurance [46].

Regarding endurance performance, studies investigating plant protein effects are limited but provide solid evidence supporting our results. Plant-based protein ingestion can improve anaerobic and aerobic capacity [10,26,41,47]. Plant-based diets may enhance endurance performance by increasing exercise capacity, modulating exercise-induced oxidative stress, and reducing inflammation [48,49]. Barnard et al. suggested that plant-based diets could improve performance and recovery in endurance sports through effects on blood flow, body composition, antioxidant capacity, systemic inflammation, and glycogen storage [50]. Further research is needed to explore the relationship between plant-based protein and endurance performance.

For MPS, numerous review studies support our findings, although our meta-analysis included only two studies comparing plant-based protein to no protein, leading to high heterogeneity and low-quality results. Goldman et al. concluded that plant-based diets exceed leucine requirements for maximal MPS stimulation, supporting daily energy needs, muscle mass, and athletic performance [51]. The high heterogeneity in our meta-analysis may stem from conflicting conclusions and measurement differences between the included studies. For instance, Oikawa et al. found that potato protein stimulates MPS at rest [11], while Davies et al. reported that fava bean protein does not improve myofibrillar protein synthesis at rest [43]. More studies are needed to provide comprehensive evidence in this field.

### 5.2. Plant-Based Protein vs. Other Types of Protein

Our meta-analysis revealed that plant-based protein does not offer greater benefits on athletic ability compared to other protein types, especially whey protein. Other protein types showed greater improvements in athletic performance and muscle strength, particularly when assessed by change value. The statistical insignificance in athletic performance presented by final value may be due to baseline differences among participants.

Several studies support these findings. On the one hand, some studies included in our meta-analysis concluded that there was no difference in improving athletic performance between plant-based protein and animal protein [2,3,4,5,6,26,40,52]. On the other hand, the International Society of Sports Nutrition’s position on sports and protein debates whether vegetarian diets are superior to omnivorous diets, with soy considered a lower-quality complete protein [53]. Plant-based proteins like soy, pea, or quinoa generally have poorer amino acid profiles than animal proteins [54,55]. Hevia-Larraín et al. found no difference in resistance training-induced adaptations between protein sources in untrained young men consuming adequate protein [56]. Previous meta-analyses have shown that animal protein tends to have a more favorable effect on lean mass compared to plant protein, especially in younger adults [57,58].

For MPS, our meta-analysis showed no significant difference between plant-based protein and other protein types. Studies support that both plant-based and animal proteins improve MPS [12,13,15,38,42]. Nichele et al. concluded that plant proteins can be nutritionally adequate alternatives to animal proteins in stimulating MPS and supporting muscle mass [49]. Kersick et al. noted that consuming an effective dose of plant-based protein can lead to similar favorable changes in amino acid uptake, MPS rates, and exercise training adaptations as those observed with animal proteins [59]. The moderate heterogeneity observed may be due to differences in measurement methods, such as MPS vs. myofibrillar protein synthesis [33,59].

## 6. Strengths and Limitations

This study was the first Bayesian meta-analysis with a systematic review to investigate the efficacy of plant-based protein on athletic ability in healthy individuals, comparing it with no protein and other types of protein. Although plant-based protein was not better than other types of protein, our meta-analysis found that it has significant benefits for athletic ability, including muscle strength, endurance performance, and MPS, in young and older people. Therefore, this study provides solid and comprehensive evidence for sports supplements and offers new material and conclusions for future studies. 

However, several limitations must be addressed. First, the small sample size and moderate heterogeneity in the meta-analysis of some outcomes degrade the quality and credibility of the results. More studies are needed to provide robust evidence. Second, the participants included both older and younger individuals, as well as recreational and elite athletes. Insufficient studies prevented an effective subgroup analysis. Third, while other protein types, particularly whey protein, seem to have better efficacy than plant-based protein, the evidence remains inconclusive and requires further investigation.

## 7. Conclusions

In conclusion, plant-based protein can improve athletic ability, including muscle strength, endurance performance, and MPS, in healthy individuals. However, plant-based protein appears to be less effective than other types of proteins, such as beef, whey, or milk protein. Small sample size and moderate heterogeneity reduced the quality and credibility of some outcomes. Therefore, more studies are needed to investigate the efficacy of plant-based protein on athletic performance and MPS. Plant-based protein supplements or diets represent a promising field in sports nutrition and merit further exploration.

## Figures and Tables

**Table 1:** The Characteristic of Included Studies (Particicpants).

Code	Study	Years	Country	Study Design	Participants	Age (M ± SD)	BMI (M ± SD)
1	Deibert	2011	Germany	RCT (Parallel)	40 (40 M/0 F)	55.7 ± 4.4	27.8 ± 2.2
2	Kouw	2022	Netherlands	RCT (Parallel)	24 (24 M/0 F)	24.5 ± 4.5	22.85 ± 2.56
3	Heijden	2024	United Kingdom	RCT (Crossover)	10 (8 M/2 F)	26 ± 6	24 ± 3
4	Jentjens	2001	United Kingdom	RCT (Crossover)	8 (8 M/0 F)	27.1 ± 7.35	NA
5	Wilkinson	2007	Canada	RCT (Crossover)	8 (8 M/0 F)	21.6 ± 0.85	NA
6	Wirth	2024	Ireland	RCT (Parallel)	113 (71 M/42 F)	59.2 ± 7.7	26.2 ± 4.9
7	Pinckaers	2022	Netherlands	RCT (Parallel)	24 (24 M/0 F)	24 ± 4	25.2 ± 3
8	Loureiro	2023	Brazil	RCT (Crossover)	12 (12 M/0 F)	NA	NA
9	Teixeira	2022	Portugal	RCT (Parallel)	40 (40 M/0 F)	NA	NA
10	Joy	2013	United States	RCT (Parallel)	24 (24 M/0 F)	21.3 ± 1.9	NA
11	Pinckaers	2024	Netherlands	RCT (Parallel)	36 (36 M/0 F)	26 ± 4	23 ± 1.93
12	West	2023	United States	RCT (Parallel)	33 (24 M/9 F)	21 ± 1	24 ± 1
13	Ghosh	2010	Malaysia	RCT (Crossover)	8 (8 M/0 F)	21.5 ± 1.1	NA
14	Lynch	2020	United States	RCT (Parallel)	61 (19 M/42 F)	NA	NA
15	Naclerio	2021	United Kingdom	RCT (Crossover)	10 (10 M/0 F)	26.8 ± 1.9	25.6 ± 4
16	Babault	2015	France	RCT (Parallel)	161 (161 M/0 F)	22 ± 3.5	23 ± 3
17	Haub	2005	United States	RCT (Parallel)	21 (21 M/0 F)	65 ± 5	28.2 ± 2.6
18	Churchward-Venne	2019	Netherlands	RCT (Parallel)	36 (36 M/0 F)	23 ± 0.4	NA
19	Candow	2006	Canada	RCT (Parallel)	24 (9 M/18 F)	NA	NA
20	Oikawa	2020	Canada	RCT (Parallel)	24 (0 M/24 F)	21 ± 3	NA
21	Bartholomae	2019	United States	RCT (Parallel)	25 (2 M/23 F)	31.2 ± 9.2	24 ± 4.7
22	Reidy	2016	United States	RCT (Parallel)	68 (68 M/0 F)	NA	25 ± 0.5
23	Davies	2022	United Kingdom	RCT (Parallel)	16 (8 M/8 F)	25 ± 4	NA
24	Laskowski	2003	Poland	RCT (Parallel)	12 (NA)	16.83 ± 0.98	NA
25	Upshaw	2016	Canada	RCT (Crossover)	8 (8 M/0 F)	21.8 ± 2.3	24.5 ± 2.6
26	Röhling	2021	United Kingdom	RCT (Parallel)	21 (16 M/7 F)	29 ± 10	23 ± 1.7
27	Bijeh	2022	Iran	RCT (Parallel)	60 (60 M/0 F)	66.53 ± 3.16	NA
28	Thomson	2016	Australia	RCT (Parallel)	125 (NA)	61.7 ± 7.9	27.5 ± 3.7
29	Moon	2020	United States	RCT (Parallel)	24 (24 M/0 F)	32.8 ± 6.7	27.2 ± 1.9
30	Berg	2012	Germany	RCT (Parallel)	30 (20 M/10 F)	24 ± 2	NA
31	Kritikos	2021	Greece	RCT (Crossover)	10 (10 M/0 F)	21 ± 1.5	24.6 ± 1.2

**Table 2:** The Characteristic of Included Studies.

Code	Study	Years	Plant-Based Protein Type	Plant-Based Protein Intake	Duration	Extracted Data
1	Deibert	2011	Soy Protein	26.7 g per Serving	12 weeks	Muscle Strength Test
2	Kouw	2022	Plant-based Protein Composed of Wheat and Chickpea flour	40 g per Serving	NA	Myofibrillar Synthesis Rate
3	Heijden	2024	MyProtein Protein beverage (39.5% pea protein, 39% brown rice protein and 21.0% canola protein)	32 g per Serving	5.5 ± 2.5 Weeks	Muscle Strength Test; Myofibrillar Synthesis Rate
4	Jentjens	2001	Wheat Protein	NA	NA	Endurance Performance Test
5	Wilkinson	2007	Soy Protein	18.2 g per Serving	≥1 Week	Myofibrillar Synthesis Rate
6	Wirth	2024	Plant-based Protein Composed of Pea and Rice Protein Mixture	23 g per day	12 Weeks	Muscle Strength Test
7	Pinckaers	2022	Potato Protein	30 g per serving	NA	Myofibrillar Synthesis Rate
8	Loureiro	2023	Pea Protein	0.5 g/kg	26 Days	Muscle Strength Test
9	Teixeira	2022	Pea Protein	NA	8 Weeks	Muscle Strength Test; Endurance Performance Test
10	Joy	2013	Rice Protein	48 g per Serving	8 Weeks	Muscle Strength Test; Endurance Performance Test
11	Pinckaers	2024	Corn Protein	30 g per Serving	NA	Myofibrillar Synthesis Rate
12	West	2023	Pea Protein	NA	NA	Myofibrillar Synthesis Rate
13	Ghosh	2010	Soy Protein	5 g per serving	NA	Endurance Performance Test
14	Lynch	2020	Soy Protein	26 g per day	12 Weeks	Muscle Strength Test
15	Naclerio	2021	Vegan-protein	30 g Per Serving	4 Weeks	Muscle Strength Test
16	Babault	2015	Pea Protein	25 g Per Serving	17 Weeks	Muscle Strength Test
17	Haub	2005	Soy Protein	0.6 g/kg	14 Weeks	Muscle Strength Test
18	Churchward-Venne	2019	Soy Protein	20 g Per Serving	NA	Myofibrillar Synthesis Rate
19	Candow	2006	Soy Protein	1.2 g/kg	6 Weeks	Muscle Strength Test
20	Oikawa	2020	Potato Protein	25 g per day	NA	Myofibrillar Synthesis Rate
21	Bartholomae	2019	Mung Bean Protein	18 g per day	8 Weeks	Muscle Strength Test
22	Reidy	2016	Soy Protein	22 g per serving	12 Weeks	Muscle Strength Test
23	Davies	2022	Fava Bean Protein	0.33 g/kg	NA	Myofibrillar Synthesis Rate
24	Laskowski	2003	Soy Protein	0.5 g/kg	4 weeks	Endurance Performance Test
25	Upshaw	2016	Soy Protein	20.1 ± 2.5 g per serving	5 weeks	Endurance Performance Test
26	Röhling	2021	Soy Protein	27.2 g per Serving	12 weeks	Endurance Performance Test
27	Bijeh	2022	Soy Protein	6.75 g per serving	12 weeks	Muscle Strength Test; Endurance Performance Test
28	Thomson	2016	Soy Protein	1.2 g/kg	12 weeks	Muscle Strength Test
29	Moon	2020	Soy protein	24 g per serving	8 weeks	Muscle Strength Test; Endurance Performance Test
30	Berg	2012	Soy protein	53.3 g per serving	6 weeks	Endurance Performance Test
31	Kritikos	2021	Soy protein	1 g/kg per day	4 weeks	Muscle Strength Test; Endurance Performance Test

**Table 3:** Summary of Bayesian and frequentist meta-analysis results for two outcomes.

Results from Bayesian Meta-Analysis										Results from Trational Frequentist Meta-Analysis				
Outcome	Intervention	Comparison	Mu.vect(SMD)	Sd.vect	95%CI	Rhat	Tau	95%CI	DIC	SMD	95%CI	I^2^	*p*	Z
Athletic Performance (Change Value)	Plant-based Protein	No protein	0.281	0.065	0.159–0.412	1.001	0.18	0.017–0.362	77.3	0.24	0.15–0.34	24%	0.00001	4.85
Athletic Performance (Final Value)			0.418	0.098	0.229–0.611	1.001	0.467	0.283–0.684	103.2	0.28	0.17–0.39	58%	0.00001	4.9

**Table 4:** Summary of Bayesian and Frequentist Meta-analysis Results for Three Outcomes.

Results from Bayesian Meta-Analysis										Results from Trational Frequentist Meta-Analysis				
Outcome	Intervention	Comparison	Mu.vect(SMD)	Sd.vect	95%CI	Rhat	Tau	95%CI	DIC	SMD	95%CI	*I* ^2^	*p*	Z
Athletic Performance (Change Value)	Plant-based Protein	Other Types of Protein Ingestion	−0.119	0.047	−0.209 to −0.028	1.003	0.076	0.003–0.192	16.2	−0.12	−0.21 to −0.03	0%	0.006	2.76
Athletic Performance (Final Value)			−0.021	0.049	−0.118 to 0.072	1.003	0.046	0.001–0.128	1.8	−0.02	−0.11 to 0.07	0%	0.66	0.44
MPS			−0.177	0.343	−0.866 to 0.491	1.001	0.743	0.116–1.704	22	−0.06	−0.53 to 0.4	54%	0.79	0.26

**Table 5:** Summary of Bayesian and frequentist subgroup meta-analysis results for four outcomes (plant-based protein vs. no protein).

Results from Bayesian Meta-Analysis										Results from Trational Frequentist Meta-Analysis				
Outcome	Intervention	Comparison	Mu.vect(SMD)	Sd.vect	95%CI	Rhat	Tau	95%CI	DIC	SMD	95%CI	*I* ^2^	*p*	Z
Muscle strength (Change value)	Plant-based Protein	No protein	0.225	0.073	0.091–0.379	1.002	0.162	0.008–0.372	46.2	0.19	0.08–0.31	23%	0.0008	3.35
Muscle strength (Final value)			0.372	0.138	0.115–0.652	1.001	0.471	0.244–0.772	41	0.4	0.15–0.66	59%	0.002	3.07
Endurance performance (Change value)			0.415	0.124	0.178–0.660	1.001	0.222	0.01–0.564	23	0.4	0.2–0.61	17%	0.0001	3.93
Endurance performance (Final value)			0.479	0.154	0.187–0.801	1.001	0.53	0.182–0.940	67.2	0.5	0.2–0.8	66%	0.001	3.24

**Table 6:** Summary of Bayesian and frequentist subgroup meta-analysis results for four outcomes (plant-based protein vs. other types of protein).

Results from Bayesian Meta-Analysis										Results from Trational Frequentist Meta-Analysis				
Outcome	Intervention	Comparison	Mu.vect(SMD)	Sd.vect	95%CI	Rhat	Tau	95%CI	DIC	SMD	95%CI	*I* ^2^	*p*	Z
Muscle strength (Change value)	Plant-based Protein	Other Types of Protein Ingestion	−0.133	0.051	−0.235 to −0.034	1.001	0.086	0.004–0.214	13	−0.11	−0.2 to −0.02	0%	0.02	2.3
Muscle strength (Final value)			−0.024	0.052	−0.125 to 0.08	1.002	0.049	0.002–0.142	−3.8	−0.02	−0.13 to 0.08	0%	0.64	0.46
Endurance performance (Change value)			−0.051	0.134	−0.312 to 0.216	1.001	0.153	0.006–0.464	6.3	−0.05	−0.28 to 0.18	0%	0.66	0.44
Endurance performance (Final value)			−0.013	0.133	−0.275 to 0.243	1.002	0.158	0.007−0.474	9.2	−0.01	−0.23 to 0.22	0%	0.96	0.05

**Table 7:** Summary of Bayesian and frequentist subgroup meta-analysis results for four outcomes (plant-based protein vs. no protein).

Results from Bayesian Meta-Analysis											Results from Trational Frequentist Meta-Analysis				
Outcome	Participants	Intervention	Comparison	Mu.vect(SMD)	Sd.vect	95%CI	Rhat	Tau	95%CI	DIC	SMD	95%CI	*I* ^2^	*p*	Z
Athletic Performance (Change Value)	Older people (Age > 55)	Plant-based Protein	No protein	0.41	0.151	0.13–0.722	1.001	0.478	0.214–0.832	35.4	0.261	0.116–0.406	64.20%	0.0001	3.52
Athletic Performance (Change Value)	Young people (Age < 55)			0.244	0.074	0.1–0.395	1.003	0.086	0.002–0.246	19.6	0.24	0.11–0.379	0%	0.0001	3.57
Athletic Performance (Final Value)	Older people (Age > 55)			0.555	0.184	0.195–0.929	1.001	0.641	0.376–1.030	30.3	0.311	0.164–0.457	76.60%	0.0001	4.15
Athletic Performance (Final Value)	Young people (Age < 55)			0.285	0.1	0.097–0.49	1.001	0.185	0.008–0.518	55.1	0.269	0.095–0.444	35.40%	0.003	3.02

## Data Availability

Full data codes of the included studies can be shared upon reasonable request from the corresponding author. All data used in this study, including graphs, codes in R, and results, have been uploaded to the OSF database for sharing. (https://osf.io/qwykg/?view_only=f891577668c4448db6adcf2958d495d9, accessed on 30 July 2024).

## Supplementary Materials

The following supporting information can be downloaded at: https://www.mdpi.com/article/10.3390/nu16162748/s1, PRISMA 2020 Checklist; Figures S1–S12: The subgroup analysis based on the types of athletic performance and ages; Table S1: Search Strategy.

## Abbreviations

MPS: muscle protein synthesis; PRO: protein; BMI: body mass index; SD: standardized deviation; CMJ: counter-movement jump; SMD: standardized mean deviation; MCMC: Markov chain Monte Carlo; μ: effect sizes; RCTs: randomized controlled trials; 1RM: one-repetition maximum; τ: heterogeneity; Rhat: convergence of Markov chains; ROB: risk of bias; ΔSD: mean change difference with corresponding standard deviation; MVC: maximum voluntary contraction; HGH: human growth hormone; M: mean; GRADE: Grading of Recommendations Assessment, Development, and Evaluation; PER: Protein Efficiency Ratio; NSCA: National Strength and Conditioning Association; PDCAAS: Protein Digestibility-Corrected Amino Acid Score; MeSH: Medical Subject Headings; PICOS: Population, intervention, comparison, outcome, and study type; PRISMA: Preferred reporting items for systematic review and meta-analysis; 95% CI: 95% confidence interval.
