# Supplementary material for: The Effect of Plant-Based Protein Ingestion on Athletic Ability in Healthy People—A Bayesian Meta-Analysis with Systematic Review of Randomized Controlled Trials
Source: Nutrients. 2024 Aug 17;16(16):2748. doi: 10.3390/nu16162748 (PMC11357476; doi:10.3390/nu16162748)
Supplement: Supplementary file 1 [file nutrients-16-02748-s001.zip › nutrients-3157188-Supplementary materials.pdf]

Supplementary Materials

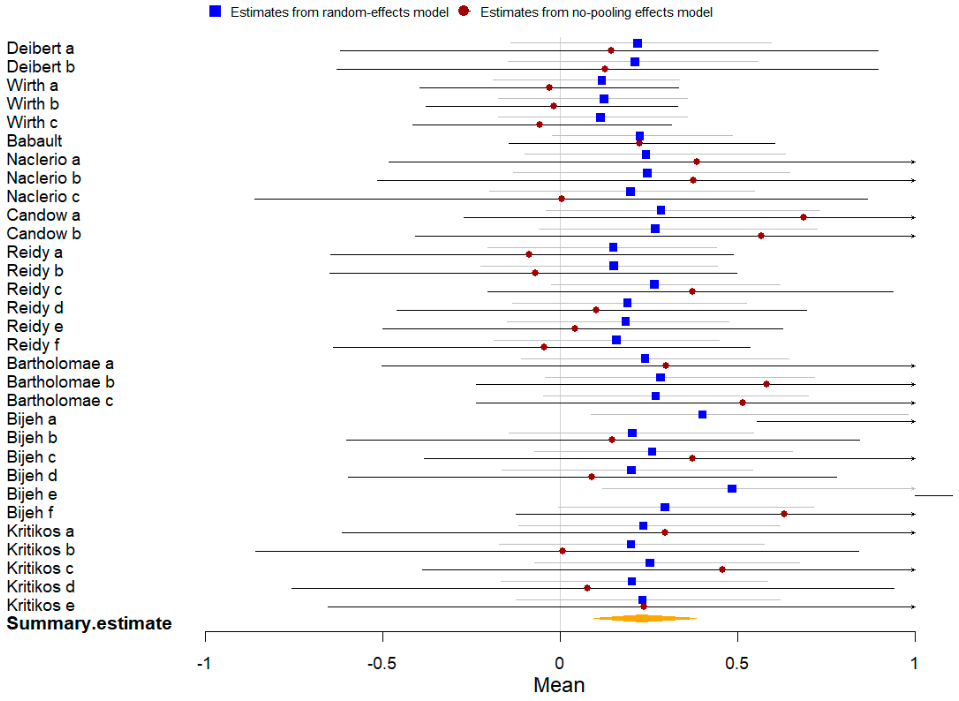

Supplementary Figure S1 The Subgroup Analysis of Muscle Strength (Plant-based Protein vs. Non-protein, Change Value)

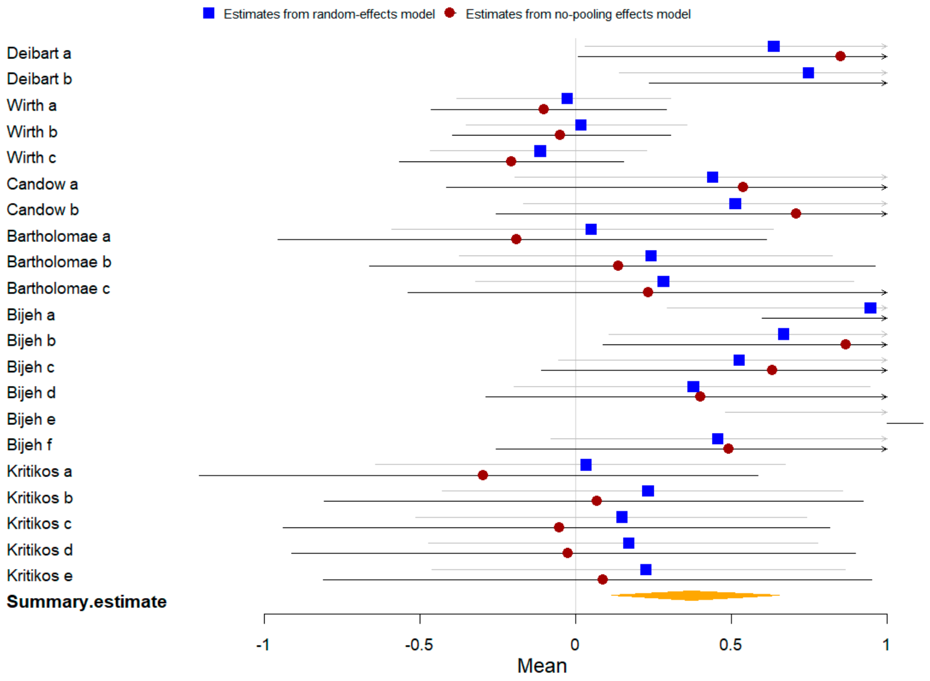

Supplementary Figure S2 The Subgroup Analysis of Muscle Strength (Plant-based Protein vs. Non-protein, Final Value)

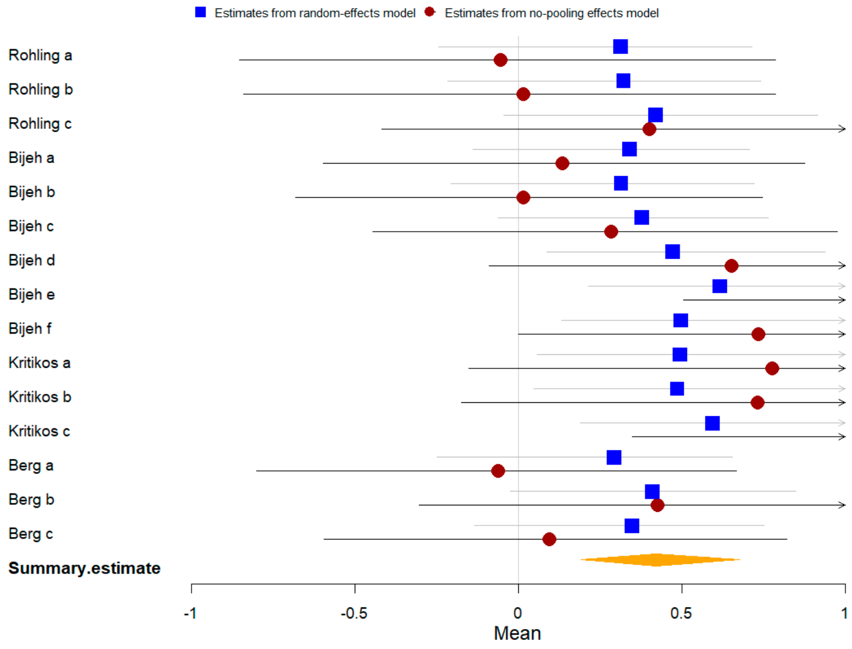

Supplementary Figure S3 The Subgroup Analysis of Endurance Performance (Plant-based Protein vs. Non-protein, Change Value)

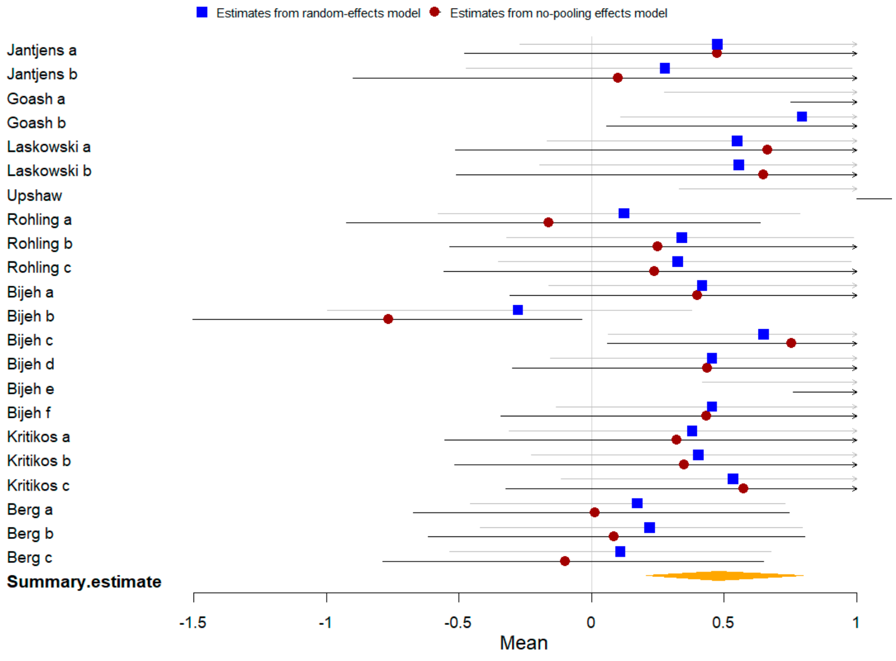

Supplementary Figure S4 The Subgroup Analysis of Endurance Performance (Plant-based Protein vs. Non-protein, Final Value)

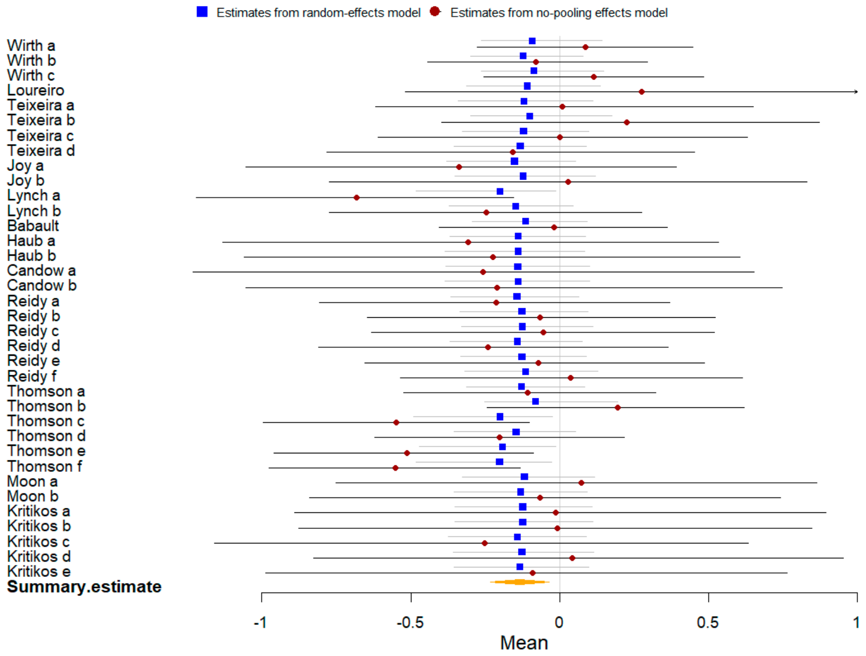

Supplementary Figure S5 The Subgroup Analysis of Muscle Strength (Plant-based Protein vs. Other types of protein, Change Value)

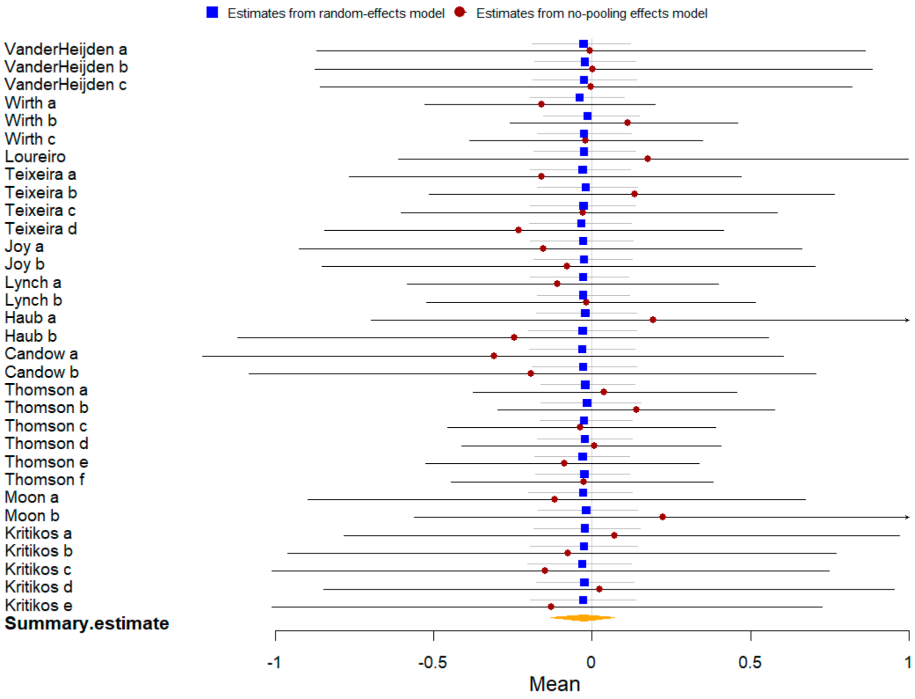

Supplementary Figure S6 The Subgroup Analysis of Muscle Strength (Plant-based Protein vs. Other types of protein, Final Value)

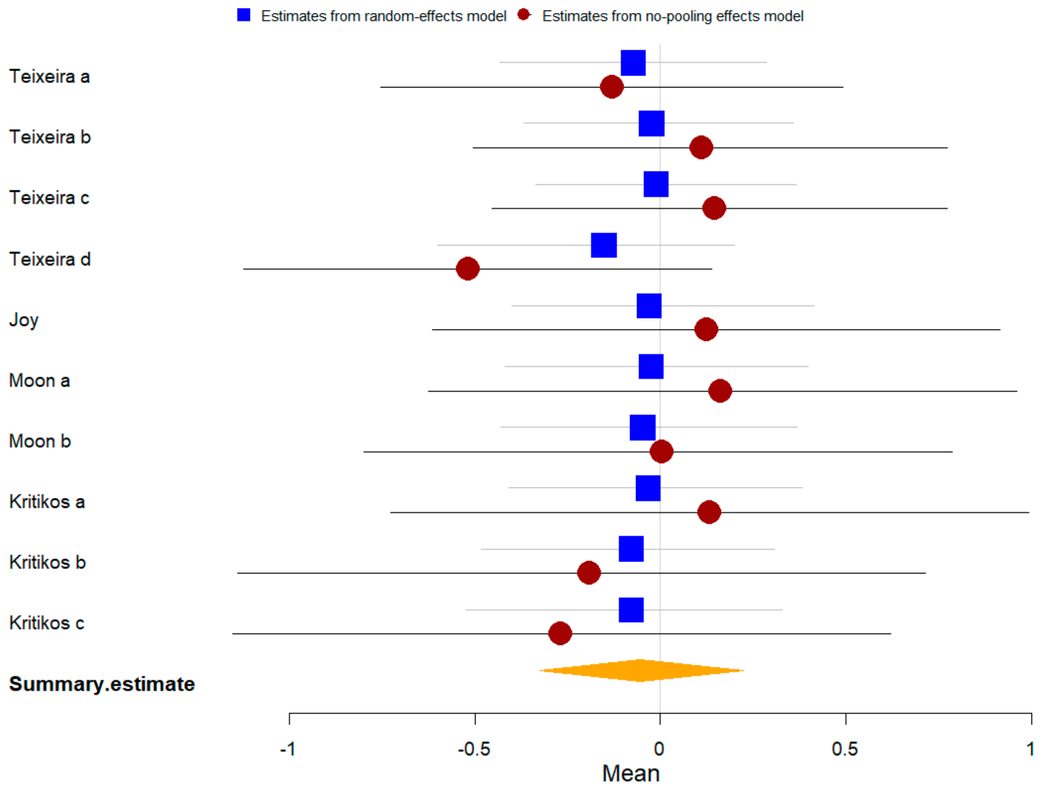

Supplementary Figure S7 The Subgroup Analysis of Endurance Performance (Plant-based Protein vs. Other types of protein, Change Value)

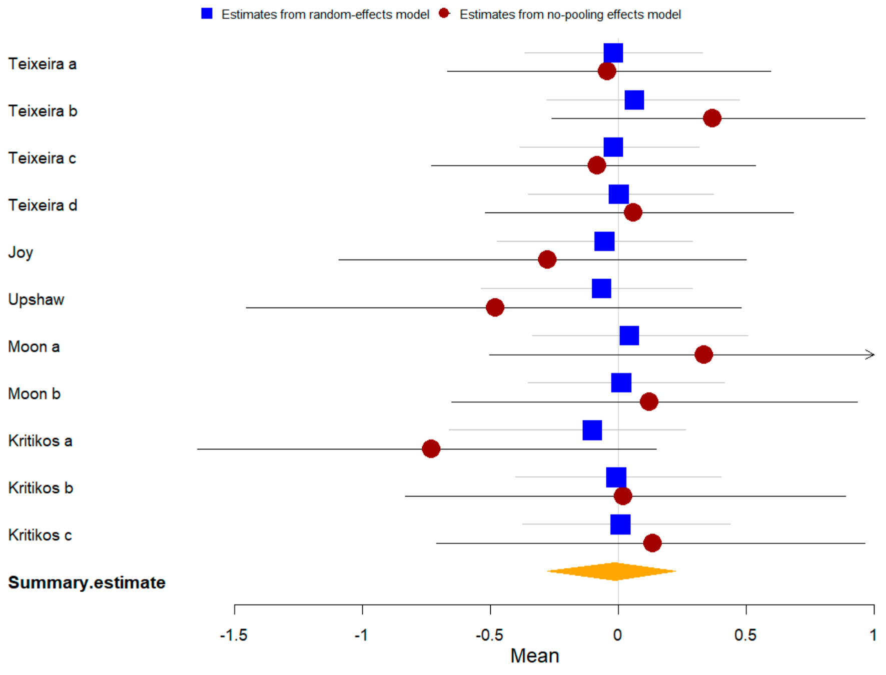

Supplementary Figure S8 The Subgroup Analysis of Endurance Performance (Plant-based

Protein vs. Other types of protein, Final Value)

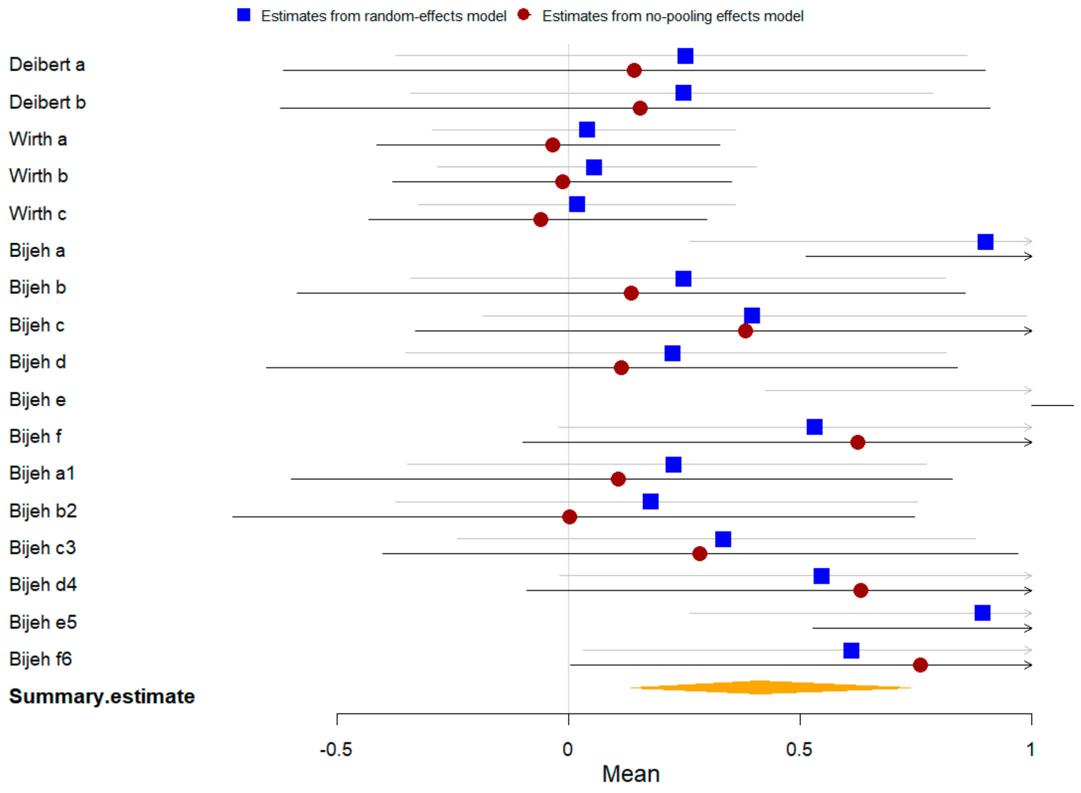

Supplementary Figure S9 The Subgroup Analysis of Older People (Change Value)

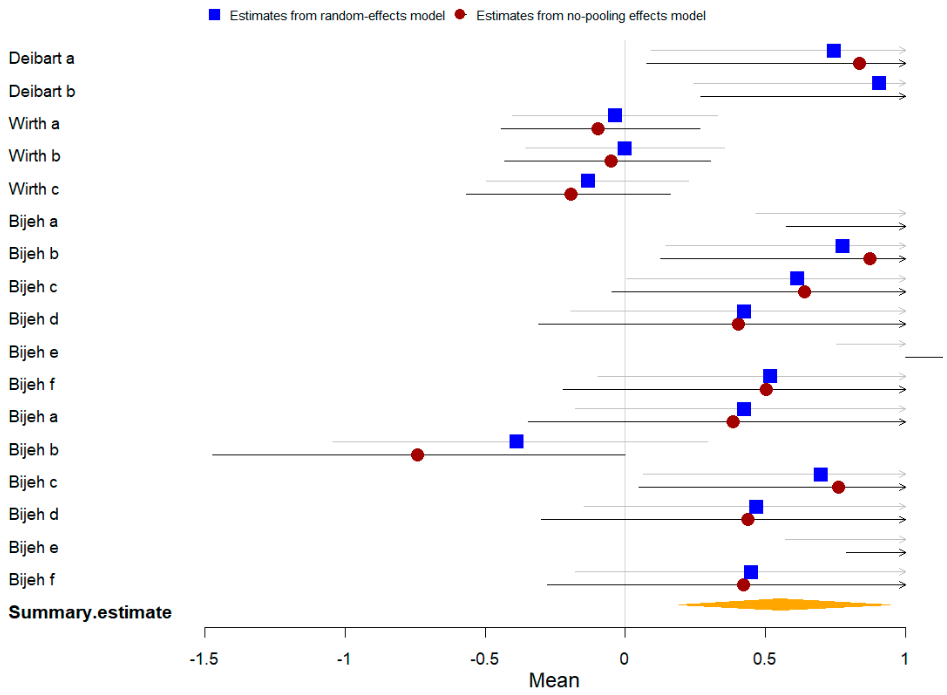

Supplementary Figure S10 The Subgroup Analysis of Older People (Final Value)

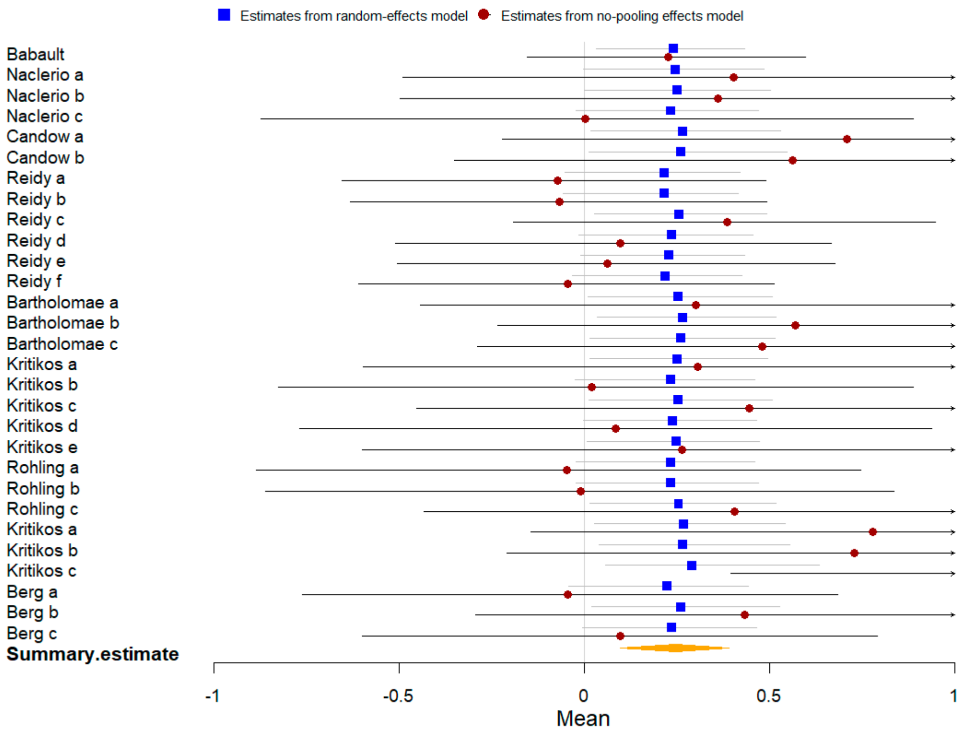

Supplementary Figure S11 The Subgroup Analysis of Young People (Change Value)

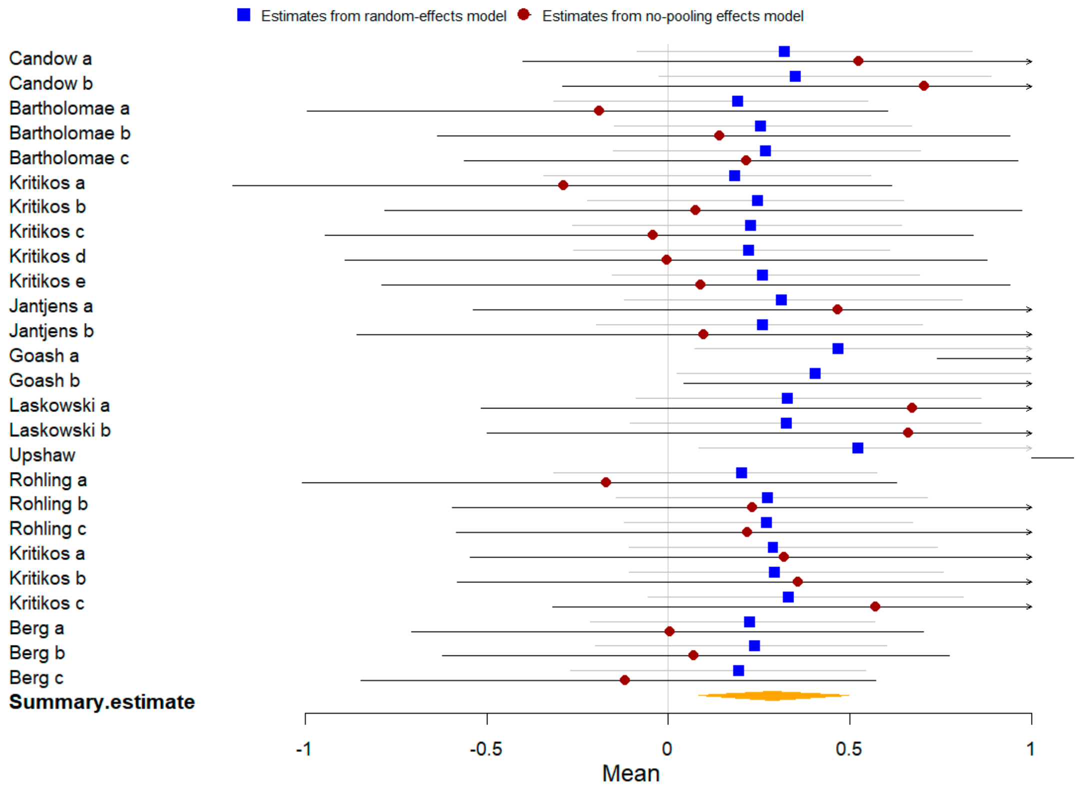

Supplementary Figure S12 The Subgroup Analysis of Young People (Final Value)

Supplementary Table S1 Search Strategies

	**Web of Science**<br>**June 25 2024**
1	Plant Protein OR Soy protein OR Pea protein OR Peanut protein OR Oat protein OR Potato Protein OR Plant protein supplements
2	Post-exercise recovery OR Athletic performance OR Sports performance OR Muscle soreness OR Resistance training OR Endurance performance OR Aerobic ability OR Anaerobic ability OR Muscle strength OR Maximum strength OR Lower body strength OR Post-exercise fatigue OR Fatigue
3	#1 AND #2
	**Pubmed**<br>**June 25 2024**
1	Soy protein OR Plant protein OR Pea protein OR Rice protein OR Peanut protein OR Potato protein OR Plant protein supplements AND Healthy adults AND Post-exercise recovery OR Athletic performance OR Sports performance OR Muscle soreness OR Resistance training OR Endurance performance OR Aerobic ability OR Anaerobic ability OR Muscle strength OR Lower body strength OR Post-exercise fatigue OR Fatigue OR Muscle injury
	**EBSCO （CINAHL）**<br>**June 25 2024**
1	plant protein OR soy protein OR pea protein OR peanut protein OR oat protein OR potato protein OR plant protein supplements
2	post-exercise recovery OR athletic performance OR sports performance OR muscle soreness OR resistance training OR endurance performance OR aerobic ability OR anaerobic ability OR muscle strength OR lower body strength OR post exercise fatigue
3	S9 AND S10

	**Scopus**<br>**June 15 2024**
1	post-exercise AND recovery OR athletic AND performance OR sports AND performance OR muscle AND<br>soreness OR resistant AND training OR endurance AND performance OR aerobic AND ability OR anaerobic AND ability OR muscle AND training OR maximum AND strength OR lower AND body AND strength OR post-exercise AND fatigue OR fatigue OR exercise AND induced AND fatigue OR muscle AND injury AND healthy AND adults AND plant AND protein OR pea AND protein OR soy AND protein OR peanut AND protein OR oat AND protein OR plant AND protein AND supplements OR potato AND protein
	**Ovid**<br>**June 25 2024**
1	Plant protein OR plant-based protein AND post exercise recovery OR athletic performance OR Sports Performances<br>OR muscle soreness OR resistance training or endurance performance or aerobic ability or anaerobic ability or muscle strength or lower body strength or post exercise fatigue AND healthy people
	**Proquest**<br>**June 25 2024**
1	(plant protein) OR (plant-based protein)
2	(post-exercise recovery) OR (athletic performance) OR (sports performance) OR (muscle soreness) OR (resistant<br>training) OR (endurance performance) OR (aerobic ability) OR (anaerobic ability) OR (muscle training) OR (maximum strength)
3	(Healthy people) NOT review OR (systematic review) OR (meta analysis)
4	[S1] AND [S2] AND [S3]
